# Bridging late-life depression and chronic somatic diseases: a network analysis

**DOI:** 10.1038/s41398-021-01686-z

**Published:** 2021-10-30

**Authors:** Federico Triolo, Martino Belvederi Murri, Amaia Calderón-Larrañaga, Davide Liborio Vetrano, Linnea Sjöberg, Laura Fratiglioni, Serhiy Dekhtyar

**Affiliations:** 1 Aging Research Center, Department of Neurobiology, Care Sciences and Society, Karolinska Institutet and Stockholm University, Stockholm, Sweden; 2 Institute of Psychiatry, Department of Neuroscience and Rehabilitation, University of Ferrara, Ferrara, Italy; 3 Centro Medicina dell’Invecchiamento, IRCCS Fondazione Policlinico Universitario “A. Gemelli”, and Università Cattolica del Sacro Cuore, Rome, Italy; 4 Stockholm Gerontology Research Center, Stockholm, Sweden

**Keywords:** Depression, Scientific community

## Abstract

The clinical presentation of late-life depression is highly heterogeneous and likely influenced by the co-presence of somatic diseases. Using a network approach, this study aims to explore how depressive symptoms are interconnected with each other, as well as with different measures of somatic disease burden in older adults. We examined cross-sectional data on 2860 individuals aged 60+ from the Swedish National Study on Aging and Care in Kungsholmen, Stockholm. The severity of sixteen depressive symptoms was clinically assessed with the Comprehensive Psychopathological Rating Scale. We combined data from individual clinical assessment and health-registers to construct eight system-specific disease clusters (cardiovascular, neurological, gastrointestinal, metabolic, musculoskeletal, respiratory, sensory, and unclassified), along with a measure of overall somatic burden. The interconnection among depressive symptoms, and with disease clusters was explored through networks based on Spearman partial correlations. Bridge centrality index and network loadings were employed to identify depressive symptoms directly connecting disease clusters and depression. *Sadness*, *pessimism*, *anxiety*, and *suicidal thoughts* were the most interconnected symptoms of the depression network, while somatic symptoms of depression were less interconnected. In the network integrating depressive symptoms with disease clusters, *suicidal thoughts*, *reduced appetite*, and *cognitive difficulties* constituted the most consistent bridge connections. The same bridge symptoms emerged when considering an overall measure of somatic disease burden. *Suicidal thoughts*, *reduced appetite,* and *cognitive difficulties* may play a key role in the interconnection between late-life depression and somatic diseases. If confirmed in longitudinal studies, these bridging symptoms could constitute potential targets in the prevention of late-life depression.

## Introduction

Late-life depression represents a major public health concern due to its impact on disability, quality of life, and health-related behaviours [1]. Compared to younger individuals, depression in older adults is presumed to have different pathophysiology and clinical presentation, with a prominent role of medical comorbidities [2–4]. Chronic somatic diseases involve neurobiological alterations, such as microvascular brain damage, autonomic, immunometabolic, or neuroendocrine dysregulation, which can have important implications for the risk of late-life depression [4]. These mechanisms coexist and interact with an array of psychological reactions to illness, that are closely related to depression, namely demoralization, anxiety, or death thoughts [4–6]. Hence, chronic somatic diseases are likely an important factor shaping the pathophysiology and clinical heterogeneity of late-life depression [7–9]. Yet, the interplay between the broad account of somatic conditions in late-life and individual depressive symptoms remains unclear.

Recently, a network approach has been increasingly adopted in psychiatric research. It enables to examine the structure of psychiatric syndromes by highlighting symptom relationships, and by identifying the most interconnected symptoms [10, 11]. The network theory of mental disorder is based on the notion that psychiatric disorders emerge from symptoms that cause and interact with each other in a reinforcing manner and via feedback loops, until they become self-sustained [10–12]. Each symptom of a mental disorder can become activated by other psychiatric symptoms or by stimuli that are external to the network. The burden of somatic diseases could be such external factor, introduced into the depressive symptom network via so-called bridge connections [10]. Such direct links between depressive symptoms and somatic disease burden may reflect either a causal process or a shared aetiological influence.

Network analysis has been previously used to visualize the structure of depressive symptoms in a large European sample of older community-dwellers, in which death wishes, depressed mood, and loss of interest were the most interconnected in the network, whereas somatic symptoms of sleep problems and loss of appetite were less interconnected [13]. Such framework has also helped uncover how clinical correlates of somatic diseases, such as pain and dyspnea, were linked to several depressive symptoms in individuals with chronic pain and chronic obstructive pulmonary disease, respectively [14, 15]. Yet, studies on older adults with multiple co-occurring diseases (i.e., multimorbidity), which detrimentally impact functional status, and increase the vulnerability to depression, are currently lacking [16–18]. Identifying whether late-life depression is associated with different somatic diseases through distinct bridge symptoms might hold clinical value for diagnostic and treatment purposes.

The aim of this study is to explore the role of somatic disease burden in the depressive symptom network of older adults. Specifically, we aim (i) to describe the structure of depressive symptoms in late life, and (ii) to identify bridge symptoms of depression directly linked to the different measures of somatic disease burden. We hypothesize that (i) system-specific clusters of somatic diseases would connect to different symptoms of the network, although we also expect some bridge connections to be shared, and (ii) that somatic symptoms of depression would emerge as especially relevant bridge symptoms between depression and somatic diseases.

## Methods

### Study population and participants

We used data from the Swedish National Study on Aging and Care in Kungsholmen (SNAC-K, www.snac-k.se), an ongoing prospective population-based cohort of individuals aged 60+ living in central Stockholm [19]. During the baseline assessments in 2001−2004, 3,363 people (73% participation rate) underwent a medical examination, neuropsychological evaluation, nurse interviews, and laboratory testing. SNAC-K has been linked to the Swedish National Patient Register, which extends participants’ clinical status with information on inpatient and outpatient care.

As part of the eligibility criteria, we excluded one participant with intellectual disability and 310 with definite, probable, or questionable dementia according to DSM-IV criteria [20]. Additional exclusions due to missing data were made as follows: ten participants who refused to undergo the medical examination and 182 with missing information on the psychiatric assessment, resulting in an analytical sample of 2,860 individuals (study population flowchart in Supplementary Fig. 1).

Written informed consent was obtained from all participants or from their next of kin in the case of cognitive impairment. SNAC-K received approval from the Ethics Committee at Karolinska Institutet and the Regional Ethical Review Board in Stockholm.

### Depressive symptoms

Psychiatric assessment was performed by trained physicians using the Comprehensive Psychopathological Rating Scale, a broad battery evaluating the presence and severity of psychiatric symptoms and observed behaviours [21]. In SNAC-K, a subset of the original version comprising 27 psychiatric features was used to assess mood, behavioural, cognitive, and somatic symptoms on a 0−6 scale of severity (i.e., absent to severe). We excluded the symptoms of *reduced sexual interest* and *morbid jealousy* because of the high missing rates (35% and 41%, respectively) and four items not pertaining to depression (*suspicion, fabrication, disinhibition, difficulty gauging social boundaries*; see Supplementary Table 1), resulting in a total of 21 symptoms.

### Somatic disease burden

Medical diagnoses in SNAC-K were based on clinical examination, medical history, laboratory data, and medication use, whereas the linkages to inpatient and outpatient clinical registers increased the accuracy of diagnostic episodes. Chronic diseases were coded according to a previously developed operationalization in SNAC-K, whereby a multidisciplinary panel of clinicians and researchers reached a consensus on the definition of chronic diseases as those with a prolonged duration, and resulting in either (1) residual disability or worsened quality of life, or (2) requirement of a long period of care, treatment, or rehabilitation [22]. As a result, 918 ICD-10 codes identifying chronic diseases were selected, and further categorized into 60 homogeneous groups in accordance with clinical criteria and relevance for old age [22].

To obtain the measures of somatic disease burden, psychiatric conditions (depression and mood disorders, schizophrenia and delusional disorders, neurotic and stress-related disorders, sleep disorders, and other psychiatric disorders) were excluded from the list of chronic disease categories. Somatic conditions were further grouped into seven clusters based on the main bodily systems: cardiovascular, metabolic, neurological, musculoskeletal, gastrointestinal, respiratory, and sensory. An unclassified cluster was also constructed, comprising diseases that were largely asymptomatic (e.g., chronic kidney disease) or could not be distinctively classified into any of the defined groups (e.g cancer, allergy; see Supplementary Table 2 for more information). The count of diseases within each cluster was used as a measure of somatic disease burden, while the total count of diseases was used as the index of the overall somatic disease burden, similar to previous studies [17].

### Statistical analyses

#### Network estimation

Analyses were based on the network approach to psychopathology [10]. In a symptom network, variables are represented as nodes connected with lines of varying thickness (i.e., edges), depending on the strength of the correlation between the nodes. The network visualization employs the Fruchterman−Reingold algorithm that places highly correlated nodes closer together and centrally in the network, while the nodes with weaker connections are positioned peripherally [23].

Network estimation was based on Spearman partial correlations: we computed the unregularized Gaussian Graphical Model using *ggmModSelect* command of the *qgraph* (version 1.6.5) package in R. Briefly, *ggmModSelect* employs an iterative process to select the optimal Gaussian Graphical Model without regularization based on the extended Bayesian information criterion (for more details, http://psychosystems.org/qgraph_1.5). Unregularized models have been suggested for samples where participants greatly outnumber the nodes [24].

Network estimation was preceded by the identification of redundant depressive symptoms, which were then merged with an averaging approach (see Supplementary Text for details) [25]. The inclusion of highly correlated symptoms in a network, a phenomenon termed topological overlap, can result in biased estimates of connections and centrality indices [26].

Three networks were estimated separately: (i) the network exploring the overall structure of depressive symptoms, (ii) the network integrating measures of disease count within each of the eight somatic clusters, and (iii) the network utilizing the overall count of somatic diseases instead.

#### Network measures

Network descriptives were examined using the following metrics: distribution degree, network density, small-worldness (we refer to the Supplementary Text for the description of the specific measures).

To identify depressive symptoms with greater connectivity, we computed the centrality index of expected influence, which for a given node is the standardized sum of all of its correlation coefficients [27]. Additionally, we used the *mgm* R package (version 1.2-9) to estimate node predictability [28], which indicates the variance in the node accounted for by its neighbouring connections [29, 30].

To detect bridge connections, i.e., those depressive symptoms that are directly connected to the different measures of somatic disease burden, we estimated the bridge centrality index of expected influence using the *networktools* package in R (version 1.2.3) [31]. This requires an a priori designation of the groups of nodes that are expected to be linked through bridge connections, which, in our case, were depressive symptoms and somatic disease clusters. The bridge’s expected influence quantifies the standardized sum of node’s edges directed to the nodes of another network, e.g., from the node of cardiovascular burden to all depressive symptoms [32]. Further, we computed the network cross-loadings, a recently developed measure that allows to estimate effect sizes of bridge connections, for which cut-off criteria have also been proposed (see Supplementary Text for details) [33, 34].

#### Sensitivity analyses

To evaluate the robustness of the obtained bridge symptoms, we computed multi-adjusted networks by adding nodes for age, sex, and education, as the partial correlations (i.e., edges in the network) are mutually adjusted for each included node [35]. Due to the scale of the variables included in the model (nominal for sex, ordinal for education), we used mixed-models analyses which can handle data on different scales (for more details, see Supplementary Text) that was carried out with the *mgm* package [28].

The stability of the network was estimated with a case-dropping bootstrap procedure using *bootnet* (version 1.4.3) [23], whereby a subset of random cases was gradually removed and the centrality measures from these reduced samples were compared with the original. The correlation stability coefficient (CS-C) was derived as the largest proportion of participants that could be randomly dropped with the correlation of centrality measures between the reduced and the original sample remaining at 0.7 or higher (a CS-C of 0.5 is indicative of a stable network).

All data preparation was conducted in STATA 16 (StataCorp, College Station, Texas, USA), while all network-related analyses were carried out in R (version 4.0.0).

## Results

### Sample characteristics

Participants excluded due to missing information on any depressiv*e* symptom (*n* = 182), were more likely to be older, more cognitively impaired, and with a higher number of chronic diseases compared to those included in the analyses (*p* < 0.01 for all, data not shown).

Table 1 reports the characteristics of the 2680 participants included in the analyses. Individuals presenting burdensome depressive symptomatology comprised 12% of the analytical sample. They were more likely to be older, female, less educated, more cognitively impaired, and to have a higher burden of somatic conditions, both in terms of overall disease count and specific clusters.

**Table 1:** Descriptive characteristics of the overall study population and according to the presence of burdensome depressive symptomatology.

	Full sample	Depressive syndrome		
		MADRS^b^ ≤ 6	MADRS > 6	*p*^a^
	*N* = 2860	*N* = 2520 (88%)	*N* = 340 (12%)	
Age, mean (SD)	73.1 (10.4)	72.7 (10.3)	75.8 (11.1)	<0.01
Sex, female %	63	62	71	<0.01
Education, %				
*Elementary*	15	15	17	<0.05
*High School*	50	49	55	
*University*	35	36	28	
MMSE^**c**^, mean (SD)	28.7 (1.6)	28.8 (1.5)	28.1 (2.2)	<0.01
Number of somatic conditions, mean (SD)	3.7 (2.3)	3.6 (2.2)	4.6 (2.5)	<0.01
Somatic disease clusters, ≥1 disease, %				
*Cardiovascular*	77	77	78	0.55
*Musculoskeletal*	29	28	35	<0.05
*Respiratory*	10	10	16	<0.01
*Neurological*	12	11	20	<0.01
*Metabolic*	64	65	60	0.08
*Gastrointestinal*	16	14	25	<0.01
*Sensory*	21	19	32	<0.01
*Unclassified*	49	48	56	<0.01

^a^Based on T test or Chi square test as appropriate.

^b^MADRS is a depressive rating scale obtained by combining 10 selected CPRS items [56]; a clinically validated cut-off of >6 was adopted to capture burdensome depressive symptomatology [57].

^c^Mini mental state examination.

Out of the 21 depressive symptoms considered, 10 redundant items were combined, resulting in a total of 16 depressive symptoms that were used in the analyses (Supplementary Table 3). The means and standard deviations of the depressive symptom scores in the total sample, and according to somatic disease burden are presented in Supplementary Table 4. Depressive symptoms with the highest mean scores were *cognitive difficulties*, *reduced sleep*, *lack of initiative,* and *anxiety*. Individuals with 2+ somatic diseases presented with significantly higher mean scores for several depressive symptoms, including *lack of initiative, anxiety, reduced appetite, cognitive difficulties*, and *suicidal thoughts*, compared to those with 0−1 conditions.

### Network of depressive symptoms

**Fig. 1:**
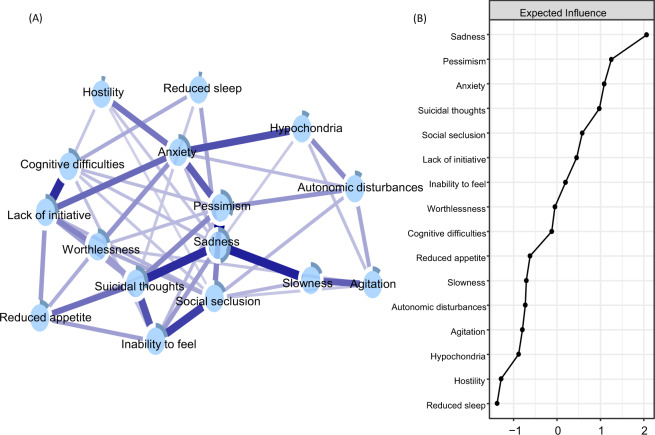
Network of depressive symptoms (panel **A**) and centrality measure of expected influence (panel **B**). Blue connections indicate positive correlations; the thickness of lines is proportional to the strength of the correlation. The darker outer circle of each node represents the predictability, i.e., the proportion of variance explained by its neighbouring nodes. Expected influence is the standardized sum of the weights of all direct connections between a specific symptom and all other symptoms.

A network of 16 depressive symptoms is shown in Fig. 1, panel A. All depressive symptoms were positively interconnected around *sadness, pessimism, anxiety*, and *suicidal thoughts*, which exhibited the highest centrality and predictability estimates (Fig. 1, panel B). The strongest connections pertained to the edges between *sadness* and *pessimism, sadness and slowness*, and *sadness* and *suicidal thoughts*. The connections between the *inability to feel* and *social seclusion*, as well as between *inability to feel* and *suicidal thoughts* were also strong, as were the edges between *cognitive difficulties* and *lack of initiative* (Supplementary Fig. 2 for correlation matrix). The network presented an indication of small-world characteristics, i.e., high clustering and short path lengths (ω index of 0.36; see Supplementary Fig. 3 and Table 5 for more network descriptives).

### Network of depressive symptoms and somatic disease burden clusters

**Fig. 2:**
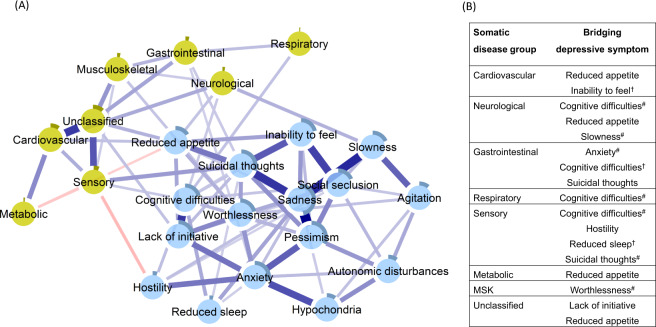
Network of depressive symptoms with nodes for system-specific clusters of somatic disease burden (panel **A**). Blue connections indicate positive correlations; red, negative correlations; thickness of lines is proportional to the strength of the correlation. The darker outer circle of each node represents the predictability, i.e., the proportion of variance explained by its neighbouring nodes. The table (panel **B**) reports bridge connections between somatic disease groups and depressive symptoms. ^#^Bridge connection preserved after including nodes for age, sex, and education. ^†^Bridge connection emergent in the network including nodes for age, sex, and education.

Figure 2, panel A, depicts the network in which the clusters of somatic disease burden were added to the network of depressive symptoms. The network presented as 24 fully interrelated nodes, with mostly positive associations, although two negative edges were also identified (see Supplementary Fig. 4 for the correlation matrix). The network exhibited an indication of being small-world (ω = 0.43; see Supplementary Fig. 3 and Table 5 for more descriptives).

**Fig. 3:**
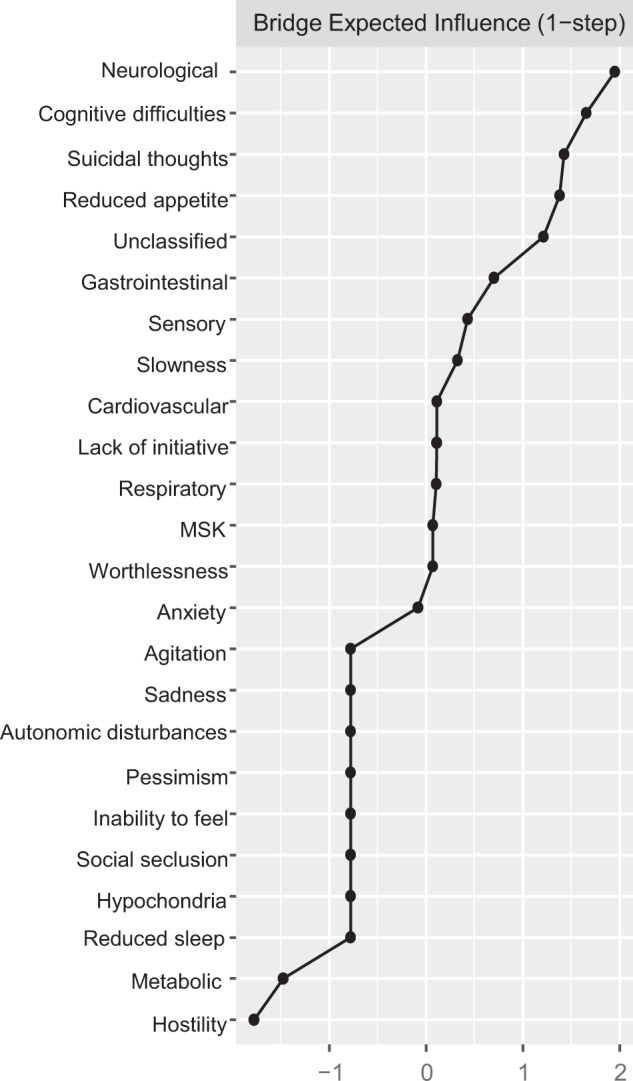
Bridge centrality measures of the expected influence of the network incorporating somatic disease clusters and depressive symptoms. Bridge expected influence expresses a node’s connectivity with the other network. The higher the centrality, the greater the connection between a node of one network (e.g., depressive symptom) with the nodes of the other network (e.g., somatic disease clusters).

Direct interconnections between depressive symptoms and somatic disease burden clusters, i.e., bridge connections, are summarized in Fig. 2, panel B. We list bridge connections from a crude network, as well as from the network including nodes for age, sex, and education. Fourteen bridge connections were identified, mostly linking somatic disease groups with symptoms on the periphery of the depressive symptoms network. *Cognitive difficulties* shared direct connections with neurological, sensory, and respiratory burden, whereas *reduced appetite* connected with neurological, cardiovascular, sensory, metabolic, and unclassified disease clusters. Conversely, *suicidal thoughts* and *anxiety*, two of the most interconnected nodes in the depressive symptoms network, presented direct links to sensory and gastrointestinal burden. Unique connections were identified between *slowness* and neurological burden, *hostility* and sensory burden, *lack of initiative* and unclassified burden, and, finally, *worthlessness* and musculoskeletal burden. The bridge centrality index of expected influence indicated that *cognitive difficulties, suicidal thoughts, a*nd *reduced appetite* exhibited the highest connectivity with the various clusters of somatic disease burden (Fig. 3). This was confirmed by the network cross-loadings, which suggested their effect sizes being substantively meaningful (0.144, 0.099, and 0.09 for *reduced appetite, cognitive difficulties,* and *suicidal thoughts*, respectively; see Supplementary Table 6 for all network loadings).

### Network of depressive symptoms and overall somatic disease burden

**Fig. 4:**
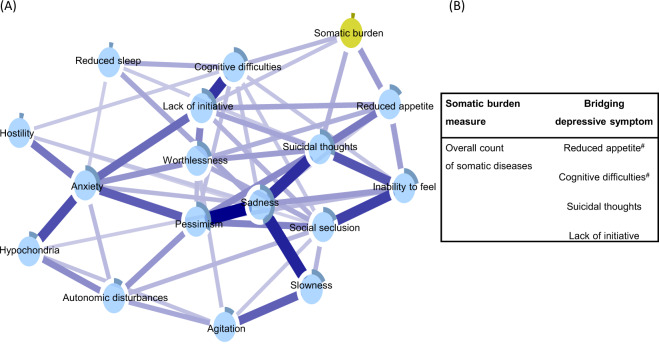
Network of depressive symptoms with a node for overall somatic burden (panel **A**). Blue connections indicate positive correlations; the thickness of lines is proportional to the strength of the correlation. The darker outer circle of each node represents the predictability, i.e., the proportion of the variance in a node explained by its neighbouring nodes. The table (panel **B**) reports bridge connections between the overall somatic burden and depressive symptoms. ^#^Bridge connection preserved after including nodes for age, sex, and education.

Figure 4, panel A, displays the depressive symptoms network integrating an overall measure of somatic burden. Parallel to the findings from the network incorporating system-specific clusters of diseases, *reduced appetite, cognitive difficulties, suicidal thoughts*, and *lack of initiative* emerged as bridge symptoms, connecting directly to the node of overall somatic burden (see Supplementary Fig. 5 for correlation matrix).

### Sensitivity analyses

Including nodes for age, sex, and education partially attenuated the bridge connections in the two networks. As summarized in Fig. 2, panel B, the following bridge connections were preserved in the network incorporating somatic disease clusters: *cognitive difficulties* and *slowness* remained connected with neurological burden, *anxiety* with gastrointestinal burden, *cognitive difficulties* and *suicidal thoughts* with sensory burden, and *worthlessness* with musculoskeletal burden. As for the association with the overall somatic burden (Fig. 4, panel B), connections with *reduced appetite* and *cognitive difficulties* persisted after adjustment.

The networks were highly stable, as all correlation stability coefficients were >0.9, indicating that the correlation of node centrality estimates between a reduced sample and the original sample was at least 0.7 after randomly excluding 90% of observations from the original sample (Supplementary Figs. 6−8).

## Discussion

Using a network approach, we examined the interconnection among depressive symptoms, as well as between depressive symptoms and different measures of somatic disease burden in a population-based sample of older adults. The network of depressive symptoms was structured around the highly interconnected symptoms of *sadness, pessimism, anxiety*, and *suicidal thoughts*. While we did uncover some unique symptom-disease cluster connections, the symptoms of *reduced appetite, suicidal thoughts*, and *cognitive difficulties* emerged as the most substantive bridge symptoms linking depression with several clusters of disease burden. Notably, these symptoms were also connected with the overall burden of somatic diseases, thus suggesting their potentially overarching influence. To our knowledge, this is the first study examining the interconnections between depression and somatic health in late life by focusing on individual depressive symptoms.

### The network of depressive symptoms in late life

This study extends the knowledge on the interconnectedness of depressive symptoms in late life. *Sadness, pessimism, anxiety*, and *suicidal thoughts* were the most interconnected symptoms of the network, while symptoms pertaining to somatic and cognitive domains, such as *reduced appetite, reduced sleep*, and *cognitive difficulties*, were less integrated. These findings are consistent with previous studies, where somatic features have shown a lower interconnectedness with other depressive symptoms [13, 36]. However, somatic and cognitive symptoms had the highest mean level scores in our sample, a finding which has also been observed in previous studies involving older adults [3, 13]. This observation has led to the hypothesis that depression in late life may be characterized by a more pronounced somatic clinical phenotype [37]. Still, we observed that the less integrated symptoms (e.g., *reduced appetite, reduced sleep*, and *cognitive difficulties*) were fully incorporated into the network through links to the more interconnected depressive symptoms, including *sadness, anxiety, suicidal thoughts*, and *lack of initiative*. Following the theory of psychological network, connections may represent sequential chains of symptoms, leading to the sustained syndrome of depression [10]. From a clinical perspective, the hypothesis posits that highly interconnected symptoms may be viewed as targets for intervention to deactivate the entire depressive network at the individual level [12]. However, evidence from network studies linking cross-sectional estimates of symptom connectivity with symptom change in individuals over time has produced mixed results [38–41]. Thus, our results should be interpreted considering the cross-sectional study design adopted here. Whether these highly interconnected symptoms are likely to be influential in some individuals’ depressive networks needs to be confirmed with longitudinal data.

### Bridge connections between depressive symptoms and somatic burden

Both more and less interconnected symptoms (i.e., *suicidal thoughts*, *reduced appetite*, and *cognitive difficulties*) were consistently linked with several measures of somatic burden covering the neurological, cardiovascular, sensory, and respiratory systems. These very same symptoms were also associated with the overall measure of disease burden. This apparent generalizability hints at the possible existence of common depressive features across different patterns of somatic diseases as well as overall multimorbidity. Overlapping symptomatology, or a complex causal structure, may underpin this association between somatic conditions and depression. Answering such questions requires future research.

*Suicidal thoughts*, a severe depressive symptom, were linked to gastrointestinal and sensory burden, as well as to the overall somatic disease burden. These findings are in line with previous studies where sensory loss was associated with suicidal ideation, and gastrointestinal disease was linked to higher rates of suicide after hospitalization [42, 43]. A recent meta-analysis concluded that individuals with physical multimorbidity were more likely to present current and future suicidal ideation [44], as well as higher suicide risk, especially when depression was comorbid [45]. In our study, the node of *suicidal thoughts* shared high connectivity with core depressive symptoms, such as *sadness* and *inability to feel*. This may suggest that older people with high clinical complexity may experience death wishes when also faced with other frequent depressive symptoms. Besides the comorbidity of depression, factors that may contribute to suicidal ideation in multimorbid individuals include functional impairment, lack of coping capacities, and chronic pain [46]. Finally, neurobiological evidence has linked suicide and suicidal ideation with impairment of body systems controlling stress response, immune function, and inflammation, which are also involved in multimorbidity [47].

We observed that *reduced appetite*, a less interconnected node, was associated with multiple somatic groups (i.e., cardiovascular, neurological, metabolic, and unclassified), as well as with the overall somatic burden. These findings are in line with the hypothesis that somatic features of depression may constitute a bridge symptom to somatic diseases, possibly due to the overlapping symptomatology. Notably, other somatic depressive symptoms in the network, such as *reduced sleep* or *autonomic disturbances*, did not emerge as bridging symptoms in any of our analyses. Older adults often experience a reduction in appetite due to conditions such as heart failure and chronic kidney disease, which can lead to the loss of weight and sarcopenia; reduced appetite can also present as a side effect of several medications (e.g., opioids, antiepileptics) [48]. It remains unclear, however, whether the experience of *reduced appetite* in the context of high somatic burden may ease the transition to depression. In our network, *inability to feel* and *lack of initiative* were the two nodes primarily connecting *reduced appetite* with the core depressive symptoms. These three depressive symptoms are thought to express impaired motivational and reward processing, which involves dopaminergic neural pathways that are typically impaired in old age [49–51].

Finally, we showed that the node for *cognitive difficulties* was less interconnected with other symptoms in the network, although it was characterized by the highest mean score in the study population. *Cognitive difficulties* displayed connections with sensory, neurological, and respiratory disease groups, as well as with the overall somatic burden. The association of neurological burden with both *cognitive difficulties* and *slowness*, exemplifies the likely symptom overlap between neurological diseases and depression, whereby depression can often precede several neurological conditions, but also manifest as one of their psychiatric symptoms. Furthermore, depression in late life is intimately linked to disrupted cognitive processes related to fronto-limbic abnormalities, often due to high somatic burden [4]. The relevance of *cognitive difficulties* is illustrated by this node’s direct connections with both central and peripheral depressive symptoms of the network, likely contributing to the sustainment of the overall depressive syndrome. Further, the association of *cognitive difficulties* with somatic groups beyond neurological diseases, as well as with the overall burden, underscores the wide-ranging implications of cognitive impairment for physical health and function that have been shown in previous studies [52–55].

### Limitations and strengths

This study has several strengths, including its population-based design, large sample size, and high participation rate (73%). The psychiatric assessment of depressive symptoms was part of an extensive examination carried out by trained physicians, whereas the clinical status was comprehensively assessed by combining medical assessment, medication reviews, and linkage to inpatient and outpatient national registries.

Several limitations need to be considered. First, we employed cross-sectional data, which hinders any conclusion about the temporality between somatic burden and depressive symptoms. Future studies should explore these associations longitudinally. Second, in networks incorporating two communities, bridge centrality may not effectively identify substantive connections, as it only provides rank-order indications. As a robustness check of our findings, we used network cross-loadings to evaluate bridge connections based on effect sizes, with *reduced appetite*, *cognitive difficulties*, and *suicidal thoughts* remaining as substantively meaningful bridges. Third, we performed basic adjustments only for age, sex, and education, which is arguably sufficient for a descriptive study that aims to be hypothesis-generating. However, future longitudinal studies may consider a broader set of potential confounders when investigating causal associations. Fourth, the data reduction procedure to combine redundant depressive items may have led to a loss of unique information pertaining to the depression assessment. To limit the loss of clinically informative nodes, we only aggregated nodes with both correlational and clinical overlap. Fifth, our classification of the somatic disease clusters may encompass high biological heterogeneity within each category. Future studies may overcome this issue by employing biological measures (e.g., inflammatory biomarkers, hyphothalamic-pituitary-adrenal axis correlates) when exploring the biological underpinning of depression in the context of somatic health. Last, the exclusion of participants with missing information on depressive symptoms may have led to the omission of older and frailer study participants.

## Conclusions

We showed that symptoms of *sadness, pessimism, anxiety*, and *suicidal thoughts* were highly interconnected in the depressive network of older adults. Furthermore, *reduced appetite*, *cognitive difficulties*, and *suicidal thoughts* consistently emerged as bridge symptoms between the depressive and the somatic disease burden networks, both for specific disease patterns, as well as for the overall burden. These findings suggest that depressive symptoms may be differentially expressed by older individuals with high clinical complexity. Future longitudinal studies are warranted to verify whether targeting these bridge symptoms in multimorbid individuals may prevent the onset of depression.

## Supplementary information

Supplementary Material

## Data Availability

Access to SNAC-K original data (http://www.snac-k.se/) is available to the scientific community upon approval provided by the SNAC-K management and maintenance committee. Applications can be submitted to Maria Wahlberg (Maria.Wahlberg@ki.se) at the Aging Research Centre, Karolinska Institutet. Code is available upon request.
