# Supplementary material for: Bridging late-life depression and chronic somatic diseases: a network analysis
Source: Transl Psychiatry. 2021 Oct 30;11:557. doi: 10.1038/s41398-021-01686-z (PMC8557204; doi:10.1038/s41398-021-01686-z)
Supplement: Supplementary file 1 — Supplementary Material [file 41398_2021_1686_MOESM1_ESM.docx]

**Supplementary Figure 1.** Flowchart of the study population


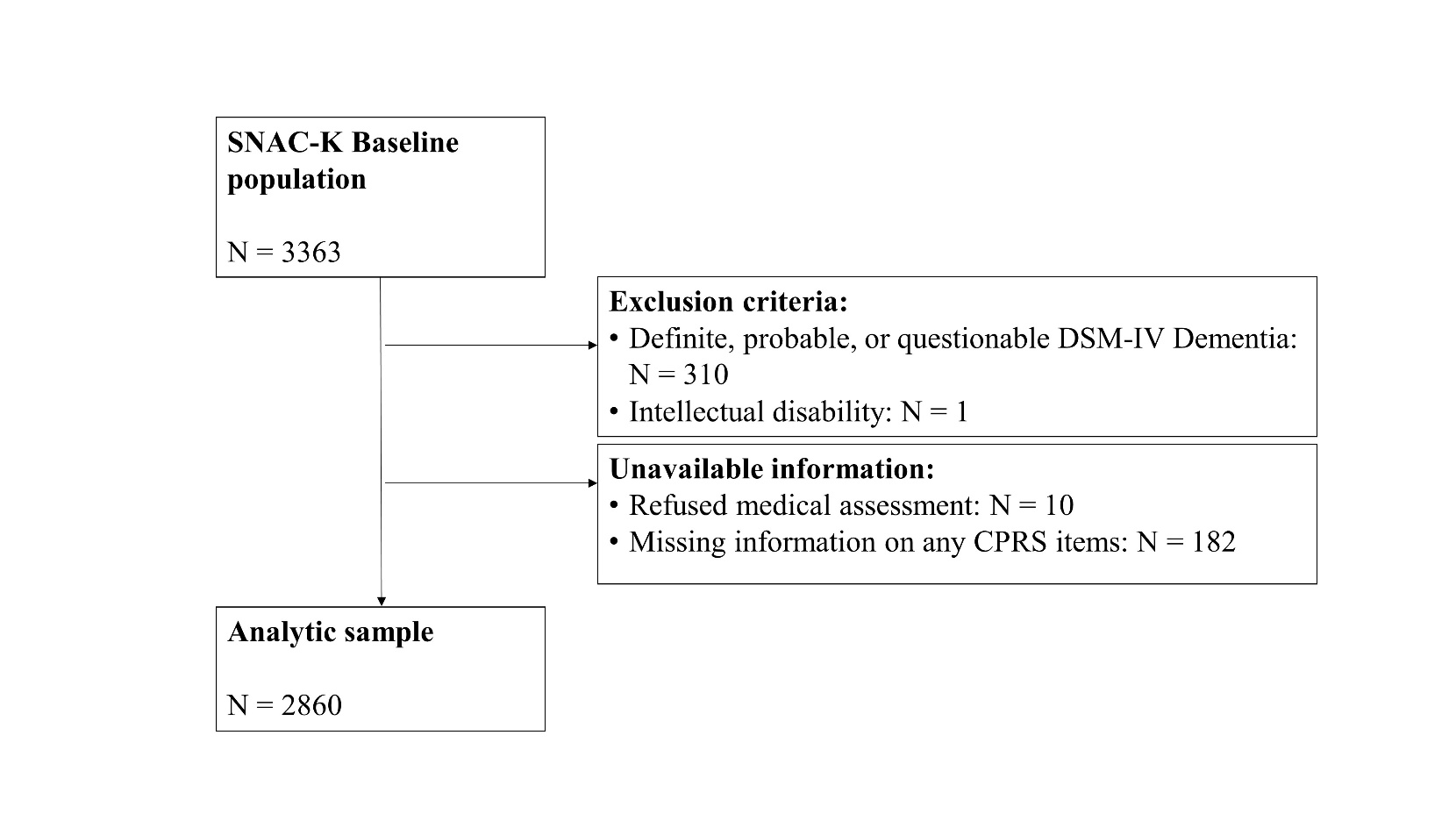


**Supplementary Table 1.** Depressive symptoms available in SNAC-K (N = 3363), including the proportion with missing data

|  | **Depressive Symptoms** | **Missing % (in total population)** |
| --- | --- | --- |
|  | Reported sadness | 4.04 |
|  | Inner tension | 4.25 |
|  | Hostility | 4.94 |
|  | Reduced sleep | 4.16 |
|  | Reduced appetite | 3.69 |
|  | Concentration difficulties | 5.20 |
|  | Hypochondriasis | 5.08 |
|  | Worrying over trifles | 5.03 |
|  | Worthlessness | 5.53 |
|  | Social seclusion | 5.06 |
|  | Indecision | 6.13 |
|  | Lassitude | 6.45 |
|  | Inability to feel | 5.41 |
|  | Pessimism | 5.17 |
|  | Failing memory | 4.91 |
|  | Autonomic disturbances | 6.24 |
|  | Suicidal thoughts | 5.14 |
|  | Observed sadness | 2.41 |
|  | Reduced speech | 2.50 |
|  | Slowness of movement | 2.23 |
|  | Agitation | 2.26 |
| **Excluded symptoms due to:**   - **Excessive missing rates (no. 22 & 23)** - **Not clinically related to depression (no. 24 to 27)** | | |
|  | Reduced sexual interest | 34.97 |
|  | Morbid jealousy | 40.65 |
|  | Difficulty gauging social boundaries | 0.06 |
|  | Disinhibition | 0.06 |
|  | Fabrication | 0.09 |
|  | Suspicion | 3.03 |

**Supplementary Table 2.** List of diseases included in the somatic burden groups and their prevalence (%), in the analytical sample.

|  | **Prevalence** |  | **Prevalence** |
| --- | --- | --- | --- |
| **Cardiovascular disease group** | | | |
| Atrial fibrillation | 8.7 | Hypertension | 70.2 |
| Bradycardias and conduction diseases | 1.8 | Ischemic heart disease | 13.9 |
| Cardiac valve diseases | 2.5 | Other cardiovascular diseases | 3.0 |
| Heart failure | 8.3 | Peripheral vascular disease | 1.5 |
| **Metabolic disease group** | | | |
| Diabetes | 8.7 | Other metabolic diseases | 1.5 |
| Dyslipidaemia | 49.3 | Thyroid disease | 10.1 |
| Obesity | 12.7 |  |  |
| **Musculoskeletal disease group** | | | |
| Dorsopathies | 6.7 | Osteoporosis | 6.6 |
| Inflammatory arthropathies | 4.2 | Other musculoskeletal and joint diseases | 6.0 |
| Osteoarthritis and degenerative joint diseases | 13.0 |  |  |
| **Respiratory disease group** | | | |
| Asthma | 6.2 | Other respiratory diseases | 1.1 |
| COPD, emphysema, chronic bronchitis | 4.4 |  |  |
| **Neurological disease group** | | | |
| Cerebrovascular disease | 6.4 | Other neurological diseases | 1.8 |
| Epilepsy | 0.6 | Parkinson and parkinsonism | 0.9 |
| Migraine and facial pain syndrome | 2.17 | Peripheral vascular disease | 1.5 |
| Multiple sclerosis | 0.1 |  |  |
| **Sensory** **disease group** | | | |
| Blindness and visual impairment | 3.2 | Glaucoma | 5.3 |
| Cataract and other lens diseases | 5.1 | Other eye diseases | 5.0 |
| Deafness, hearing impairment | 9.2 |  |  |
| **Gastrointestinal disease group** | | | |
| Chronic liver diseases | 0.2 | Esophagus, stomach and duodenum diseases | 4.5 |
| Chronic pancreas, biliary tract and gallbladder diseases | 1.5 | Inflammatory bowel diseases | 0.9 |
| Colitis and related diseases | 9.9 | Other digestive diseases | 0.6 |
| **Unclassified disease group** | | | |
| Allergy | 1.7 | Ear, nose, throat diseases | 0.9 |
| Anemia | 10.7 | Haematological neoplasms | 0.8 |
| Autoimmune disorders | 4.5 | Other genitourinary diseases | 2.5 |
| Blood and blood forming organ diseases | 0.5 | Other skin diseases | 0.1 |
| Chromosomal abnormalities | 0 | Prostate diseases | 4.4 |
| Chronic infectious diseases | 0.4 | Solid neoplasms | 9.1 |
| Chronic kidney diseases | 32.4 | Venous lymphatic diseases | 0.7 |
| Chronic ulcer of the skin | 0.6 |  |  |

**Supplementary Table 3.** List of depressive symptoms obtained after node reduction.

|  | **Original depressive symptoms** | **Derived variable after node reduction procedure** | |
| --- | --- | --- | --- |
|  | Observed sadness | Sadness | 1 |
|  | Reported sadness |  |  |
|  | Inner tension | Anxiety | 2 |
|  | Worrying over trifles |  |  |
|  | Hostility | Hostility | 3 |
|  | Reduced sleep | Reduced sleep | 4 |
|  | Reduced appetite | Reduced appetite | 5 |
|  | Concentration difficulties | Cognitive difficulties | 6 |
|  | Failing memory |  |  |
|  | Hypochondria | Hypochondria | 7 |
|  | Worthlessness | Worthlessness | 8 |
|  | Social seclusion | Social seclusion | 9 |
|  | Indecision | Lack of initiative | 10 |
|  | Lassitude |  |  |
|  | Inability to feel | Inability to feel | 11 |
|  | Pessimism | Pessimism | 12 |
|  | Autonomic disturbances | Autonomic disturbances | 13 |
|  | Suicidal thoughts | Suicidal thoughts | 14 |
|  | Reduced speech | Slowness | 15 |
|  | Slowness of movement |  |  |
|  | Agitation | Agitation | 16 |

**Supplementary Table 4.** Mean and standard deviation (SD) of depressive symptom scores in the total sample and according to somatic disease burden.

| **Depressive Symptoms** | **Total Sample** | **Somatic burden** | | *p** |
| --- | --- | --- | --- | --- |
|  |  | 0-1 disease | 2+ diseases |  |
| Anxiety | 0.43 (0.75) | 0.35 (0.65) | 0.44 (0.77) | <0.01 |
| Hostility | 0.30 (0.78) | 0.32 (0.80) | 0.30 (0.77) | 0.63 |
| Reduced sleep | 0.49 (1.04) | 0.45 (1.02) | 0.49 (1.04) | 0.42 |
| Reduced appetite | 0.19 (0.66) | 0.07 (0.40) | 0.22 (0.70) | <0.01 |
| Cognitive difficulties | 0.64 (0.81) | 0.52 (0.74) | 0.66 (0.82) | <0.01 |
| Hypochondria | 0.12 (0.46) | 0.11 (0.47) | 0.12 (0.45) | 0.73 |
| Worthlessness | 0.15 (0.54) | 0.09 (0.39) | 0.17 (0.56) | <0.01 |
| Social seclusion | 0.28 (0.70) | 0.26 (0.67) | 0.28 (0.70) | 0.56 |
| Lack of initiative | 0.42 (0.75) | 0.28 (0.57) | 0.45 (0.77) | <0.01 |
| Inability to feel | 0.10 (0.44) | 0.06 (0.34) | 0.11 (0.46) | <0.01 |
| Pessimism | 0.24 (0.64) | 0.18 (0.58) | 0.25 (0.65) | <0.05 |
| Autonomic disturbances | 0.29 (0.76) | 0.30 (0.74) | 0.29 (0.76) | 0.69 |
| Suicidal thoughts | 0.15 (0.53) | 0.06 (0.39) | 0.16 (0.55) | <0.01 |
| Sadness | 0.17 (0.48) | 0.13 (0.43) | 0.17 (0.49) | 0.07 |
| Slowness | 0.06 (0.29) | 0.05 (0.28) | 0.06 (0.29) | 0.73 |
| Agitation | 0.04 (0.27) | 0.02 (0.21) | 0.04 (0.28) | 0.13 |

*based on T test or Chi square test; each depressive symptom is assessed on a 0-6 scale


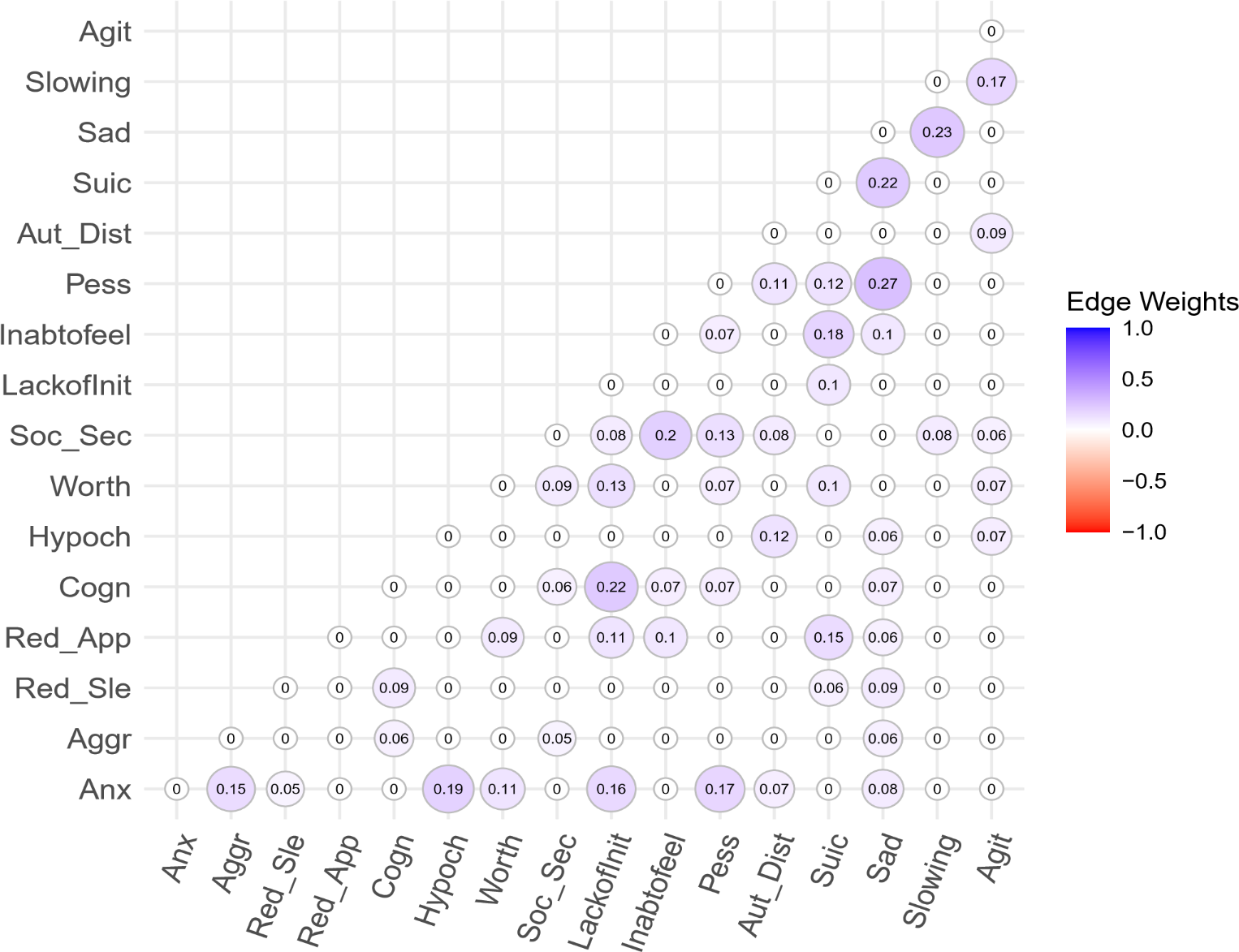
**Supplementary Figure 2.** Correlation coefficients of network with only depressive symptoms (Figure 1 in main text).

**Supplementary Figure 3.** Plot of degree of connections for the network of depressive symptoms (Panel A), as well as the network incorporating depressive symptoms and somatic diseases (Panel B).


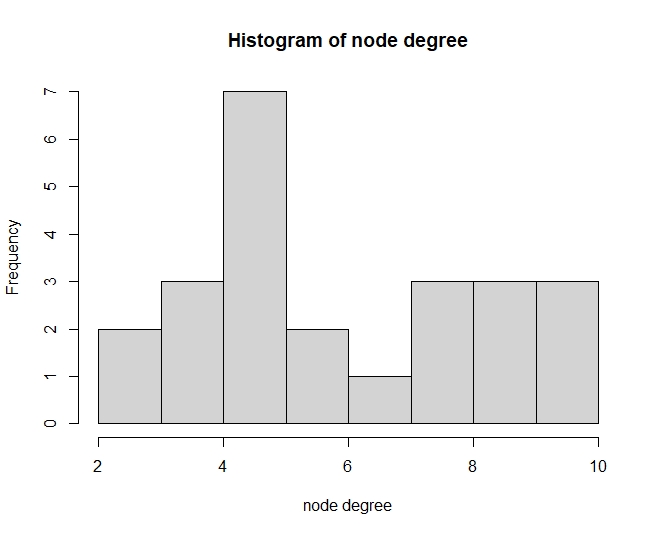

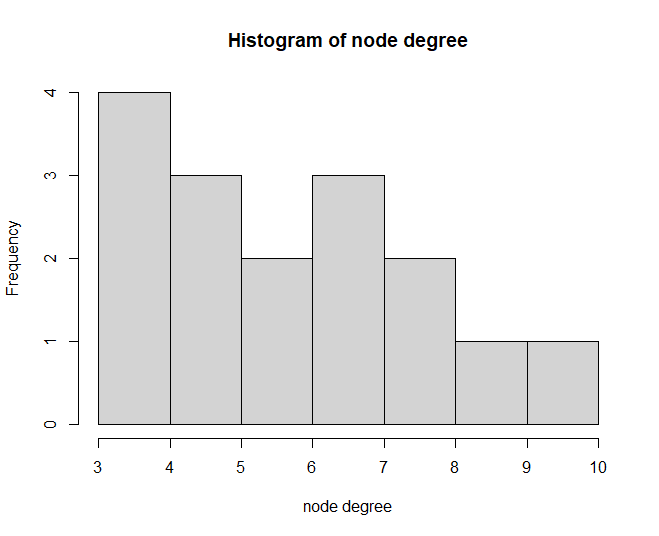


A

B

| **Measure** | **Score** | |
| --- | --- | --- |
|  | *Network of depressive symptoms* | *Network of depressive symptoms and somatic diseases* |
| Network density, computed as actual connections/potential connections ratio | 0.408 | 0.275 |
| Edges’ range | Lowest: 0.05  (Reduced sleep/Anxiety)  Highest: 0.27 (Pessimism/Sadness) | Lowest: -0.07 (Hostility/Sensory)  Highest: 0.27 (Pessimism/Sadness) |
| Small-world index ω^1^ | 0.36 | 0.43 |
| Global average shortest path length | 1.62 | 1.85 |
| Clustering coefficient | 0.67 | 0.62 |

**Supplementary Table 5**. Descriptive measures of the network with depressive symptoms only, and with somatic diseases.

**
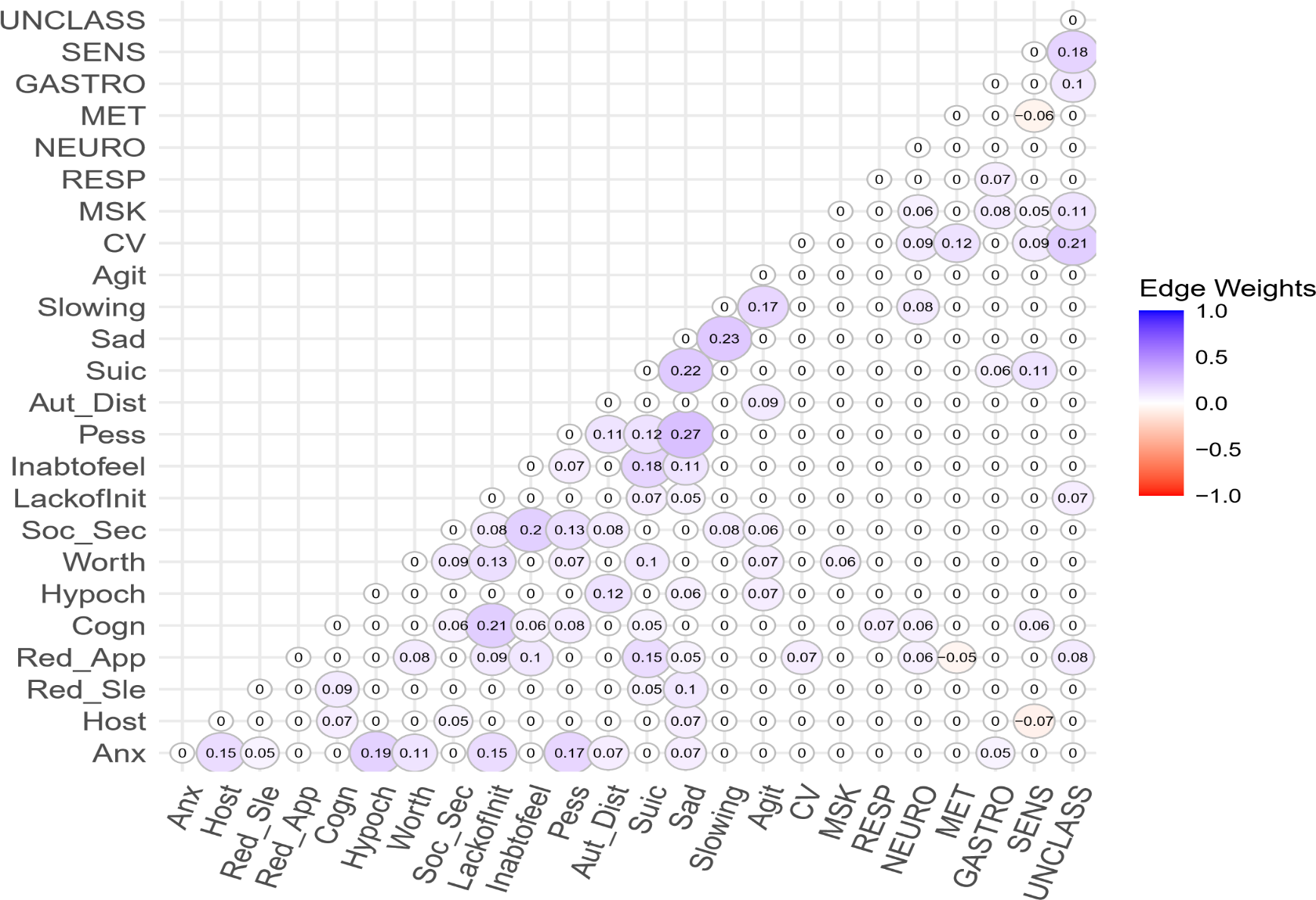
Supplementary Figure 4.** Correlation coefficients of network with depressive symptoms and somatic disease groups (Figure 2 in main text).

**Supplementary Table 6.** Network loadings of each item of the network with depressive symptoms and the overall somatic disease burden (Figure 4 in the main text). Cross-loadings (i.e. nodes’ network loading to the other community) are in bold.

|  | Depressive symptoms  community | Somatic burden  community |
| --- | --- | --- |
| Sadness | 0.358 | **0** |
| Pessimism | 0.299 | **0** |
| Anxiety | 0.281 | **0.028** |
| Suicidal Thoughts | 0.275 | **0.09** |
| Social Seclusion | 0.243 | **0** |
| Lack of Initiative | 0.23 | **0.036** |
| Inability to feel | 0.212 | **0** |
| Worthlessness | 0.191 | **0.035** |
| Cognitive Difficulties | 0.185 | **0.099** |
| Reduced Appetite | 0.139 | **0.144** |
| Slowness | 0.139 | **0.045** |
| Autonomic Disturbances | 0.139 | **0** |
| Observed Agitation | 0.133 | **0** |
| Hypochondria | 0.126 | **0** |
| Hostility | 0.098 | **-0.041** |
| Reduced Sleep | 0.086 | **0** |
|  |  |  |
| Unclassified | **0.044** | 0.325 |
| Cardiovascular | **0.02** | 0.278 |
| Sensory | **0.07** | 0.206 |
| MSK | **0.019** | 0.164 |
| Gastrointestinal | **0.033** | 0.133 |
| Metabolic | **-0.015** | 0.099 |
| Neurological | **0.06** | 0.08 |
| Respiratory | **0.019** | 0.037 |

**
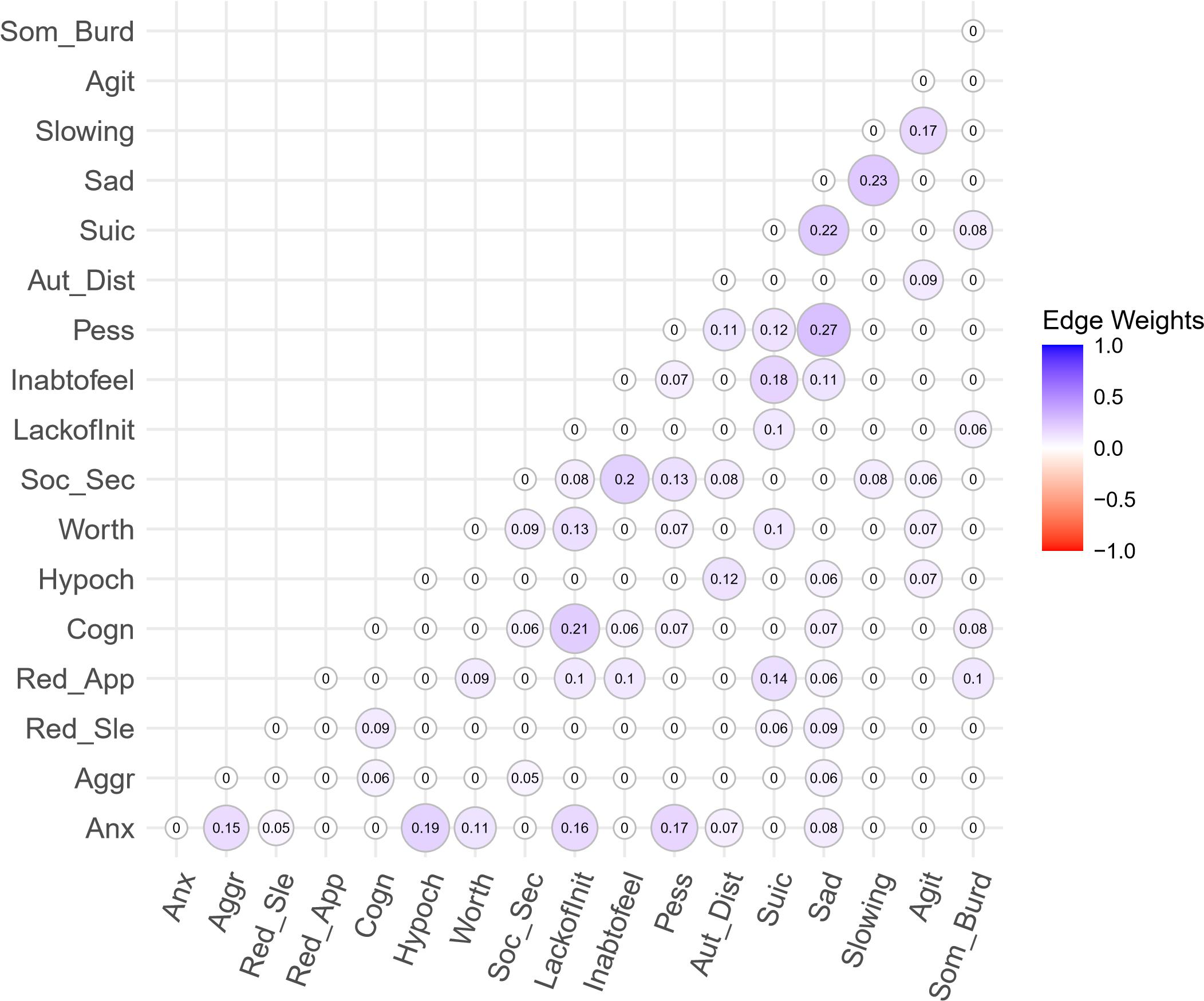
Supplementary Figure 5.** Correlation coefficients of network of depressive symptoms and overall somatic burden (Figure 4 in main text).

**Supplementary Figure 6.** Stability of centrality index (Expected Influence) by case dropping subset bootstrap for the network with only depressive symptoms (Figure 1 in the main text)

−1.0

−0.5

0.0

0.5

1.0

30

%

20

%

10

%

Sampled cases

Average correlation with original sample

**Supplementary Figure 7**. Stability of centrality index (Expected Influence) by case dropping subset bootstrap for the network with depressive symptoms and somatic disease groups (Figure 2 in the main text)

−1.0

−0.5

0.0

0.5

1.0

30

%

20

%

10

%

Sampled cases

Average correlation with original sample

**Supplementary Figure 8.** Stability of centrality index (Expected Influence) by case dropping subset bootstrap for the network with depressive symptoms and the overall somatic disease burden (Figure 4 in the main text)

−1.0

−0.5

0.0

0.5

1.0

30

%

20

%

10

%

Sampled cases

Average correlation with original sample

**Supplementary Text**

**Data reduction procedure**

Networks are visual representation of correlation between nodes. The introduction of highly correlated items in the network is discouraged due to their topological overlap. A suggested solution is to reduce the total number of nodes by aggregating those variables that present excessive content overlap. In this study we applied the *node.redundant* function from the EGAnet R package (version 0.9.6) ^2^, which identified redundant items in the network based on the weighted topological overlap ^3^. Then, the *node.redundant.combine* function merged the selected items by averaging them after approval from the researcher based on subject matter knowledge ^2^. As we aimed to merge only those symptoms exhibiting both correlational and substantive overlaps, clinical judgement was used to evaluate the aggregations proposed by the algorithm. For instance, observed and reported sadness were merged into a single item, sadness. Data reduction was performed for ten items (Supplementary Table 3), resulting in a total of 16 depressive symptoms that were ultimately used in the analysis: sadness, anxiety, reduced sleep, reduced appetite, cognitive difficulties, hypochondria, worthlessness, social seclusion, lack of initiative, inability to feel, pessimism, autonomic disturbances, suicidal thoughts, hostility, slowness, agitation.

**Network descriptives**

*Network density.* Proportion of number of actual connections over the total number of potential connections.

*Small-world index* ω*.* Quantatitive measure of the tendency of a network to present small-world characteristics, i.e. presence of areas of highly clustered nodes within the network. This index takes into account both network clustering (proportion of edges between the neighbors of a node relative to the total number of possible connectons between neighbors) and path length (distance between nodes in the network, estimated as the average of the shortest distances between all possible node pairs)^1^ . No explicit range or cut-off for small-worldness has been so far established, but simulations indicate that the small-world region spans the range −0.5 ≤ω ≤0.5^1^.

*Degree distribution.* The number of connections a node in a network has to the other nodes.

**Network loadings**

Network loadings are defined as the standardized sum of connection weights between each node and other nodes within its community (dominant loading) and nodes belonging to other communities (cross-loadings)^4, 5^. Thus, cross-loadings convey information on bridge connections, as they express a node’s contribution to other network communities ^4^. Further, they provide bridge connections with effect size, which can later be compared to proposed guidelines (small (0.15), moderate (0.25), and large (0.35))^5^. However, since cross-loadings generally tend to be smaller than 0.15, scores ≥ 0.10 have been suggested to be considered as substantively meaningful^4^.

**Adjusted network**

As sentivity analyses, we estimated the two networks with somatic disease burden (Figure 2 and 3 from the main text) with adjustment for age, sex and education. To do so, we added such nodes to the networks since the resulting connections were computed as partial correlations. Spefically, we employed the *mgm* R package, which allows to handle different types of variables. In order to reduce the chance of false-positive results, *mgm* operates a regularization through the least absolute shrinkage and selection operator (LASSO) ^6^. Briefly, a sparse network is selected by optimization of the extended Bayesian Information Criterion (EBIC), which is controlled by the parameter *λ* ^6^. Following the example of a previous study investigating the relations between depressive symptoms and inflammatory biomarkers, we set *λ* to 0 in order to enhance sensitivity towards the bridge connections between somatic burden and depressive symptoms ^7^. Such bridge associatons are in fact more likely to be smaller in magnitude compared to those between depressive symptoms, and therefore will be more more affected by the shrinkage operated by LASSO ^6^.

**REFERENCES**

1. Telesford QK, Joyce KE, Hayasaka S, Burdette JH, Laurienti PJ. The Ubiquity of Small-World Networks. *Brain Connect* 2011; **1**(5)**:** 367-375.

2. Golino H, Christensen, A. P. EGAnet: Exploratory Graph Analysis -- A framework for estimating the number of dimensions in multivariate data using network psychometrics. R package version 0.9.5 edn2020.

3. Gysi DM, Voigt A, Fragoso TdM, Almaas E, Nowick K. wTO: an R package for computing weighted topological overlap and a consensus network with integrated visualization tool. *BMC Bioinformatics* 2018; **19**(1)**:** 392.

4. Christensen AP, Garrido, L. E., & Golino, H. What is bridge centrality? A comment on Jones, Ma, and McNally (2019). *PsyArXiv* 2021.

5. Christensen AP, Golino H. On the equivalency of factor and network loadings. *Behav Res Methods* 2021; **53**(4)**:** 1563-1580.

6. Epskamp S, Fried EI. A tutorial on regularized partial correlation networks. *Psychol Methods* 2018; **23**(4)**:** 617-634.

7. Fried EI, *et al*. Using network analysis to examine links between individual depressive symptoms, inflammatory markers, and covariates. *Psychol Med* 2019**:** 1-9.
